# Responding to a protracted tuberculosis outbreak: lessons from multiple rounds of investigation in a Chinese boarding school

**DOI:** 10.1080/07853890.2026.2635885

**Published:** 2026-03-02

**Authors:** Jing Mao, Qingyan Wu, Kunyang Wu, Lina Zhao, Jun Li, Zhili Shan, Lingqiong Mao, Hante Lin, Zhen Zeng, Bin Chen

**Affiliations:** a Department of Tuberculosis and Acquired Immune Deficiency Syndrome Control and Prevention, Wenzhou Center for Disease Control and Prevention (Wenzhou Health Supervision Institution), Wenzhou, China; b Department of Infectious Disease Control and Prevention, Yueqing Center for Disease Control and Prevention (Yueqing Health Supervision Institution), Wenzhou, China; c Department of Tuberculosis Control and Prevention, Zhejiang Provincial Center for Disease Control and Prevention, Hangzhou, China

**Keywords:** Multi-semester tuberculosis outbreak, investgation and control, contact screening

## Abstract

**Purpose:**

This study analysed a multi-semester pulmonary tuberculosis (PTB) cluster outbreak in a Chinese boarding school to provide evidence for future epidemic control.

**Methods:**

Contacts were screened via symptoms, infection tests and chest radiography. Screening expanded progressively from close contacts to same-floor contacts, then all students and staff. Whole-genome sequencing (WGS) with single nucleotide polymorphism (SNP) and bioinformatics analysis was used for lineage classification, transmission clustering (≤12 SNPs defining a cluster) and drug resistance prediction.

**Results:**

From 2020 to 2022, 20 students were diagnosed with PTB, half laboratory-confirmed. Most cases clustered in class 16 and were epidemiologically linked to the primary case (case 0), who had household PTB exposure. Case 0 and case 1 had diagnostic delays exceeding 3 and 6 months, respectively. WGS of five isolates (case 1, 3, 4, 9 and 10) collected over three semesters showed all belonged to lineage 2 and differed by ≤12 SNPs, confirming the same transmission chain. The infection rate in class 16 (46.34%) was significantly higher than other case classes (19.05%) and classes without cases (8.27%) (*χ*^2^ = 61.169, *p* < 0.001). No new cases were detected during a one-year follow-up of students involved in the outbreak after the final round of screening, nor among household contacts of all cases followed up to the present.

**Conclusions:**

Lack of entry health examinations facilitated the outbreak. Delayed diagnosis, incomplete contact screening and absence of preventive treatment led to cross-semester persistence. The infection rate disparity confirms class 16 as the outbreak epicentre. Improving community case management, extending contact follow-up and enhancing cluster outbreak measures are recommended to prevent future outbreaks.

## 1. Background

Tuberculosis (TB), primarily manifested as pulmonary tuberculosis (PTB) and caused by *Mycobacterium tuberculosis* (MTB), remains a major global public health issue. Global estimates by the World Health Organization (WHO) indicate 10.7 million incident cases of TB in 2024, including 1.2 million (11%) among children and adolescents [1]. As a high-burden country, China recorded 21.9 million cumulative PTB notifications from 1997 to 2023 [2], with students accounting for approximately 5% of all notified cases nationally [3], underscoring the importance of TB control in educational settings.

Pulmonary tuberculosis, with its prolonged incubation and non-specific symptoms, facilitates outbreaks in crowded school settings [4]. Prolonged exposure among students and staff often precedes case detection, leading to clustered outbreaks that disrupt education and pose substantial health risks. In response, the WHO strongly recommends systematic contact investigation for children and adolescents, which includes rigorous screening of household and close contacts with longitudinal follow-up to effectively interrupt transmission chains [5]. However, there is a gap between these international recommendations and the operational focus of many school-based outbreak investigations. *The Guidelines for Tuberculosis Prevention and Control in Chinese Schools* (hereafter ‘*the Guidelines*’), for instance, primarily emphasize intra-school interventions, with limited directive on the systematic investigation of community/household contacts or mandated long-term (>1 year) follow-up [6]. This common practice results in outbreak reports that are typically confined to a single academic semester, lacking the temporal scope to capture delayed cases or cross-setting transmission chains [7,8]. It remains unclear how extended, multi-semester follow-up shapes our understanding of outbreak magnitude and transmission.

To address this, we conducted a retrospective analysis combined with long-term prospective follow-up of a boarding school TB cluster. Our work reconstructed transmission chains from a household source through the 2020–2022 school outbreak and has maintained ongoing active monitoring of household contacts to date. This integration of longitudinal epidemiological and molecular data allows for a more complete reconstruction of the transmission network, underscoring the value of extended, integrated investigations for outbreak control.

## 2. Investigation and results

### 2.1. Background information and definitions

This outbreak occurred in a public boarding high school comprising 55 classes across grades 10–12. The index case was reported in June 2021 within the 11th grade, encompassing 18 classes, 813 students and 70 teachers. Students resided exclusively on campus in six-bed dormitories. The school clinic employed four full-time doctors, and dormitory or classroom sanitation was maintained. However, persistent air-conditioning use with closed doors or windows during warmer months resulted in inadequate ventilation. School health monitoring was inconsistently implemented, including daily student health checks and illness-related absence documentation. Tuberculosis screening for incoming students was omitted.

Cases were categorized as clinically diagnosed or laboratory-confirmed [9]. Clinically diagnosed cases met either of the following criteria despite negative bacteriological results: exclusion of other pulmonary diseases through diagnostic treatment and follow-up observation, or radiographic evidence suggestive of TB accompanied by TB-related clinical symptoms or positive MTB infection testing. Laboratory-confirmed cases required detection of MTB through sputum smear microscopy, mycobacterial culture or GeneXpert MTB/RIF assay. Latent tuberculosis infection (LTBI) was defined as positive MTB infection test results [tuberculin skin test (TST) moderate or stronger positivity, the ESAT-6/CFP-10 fusion protein-based skin test (TBST) positivity or interferon-gamma release assay (IGRA) positivity] without evidence of active TB disease [10].

### 2.2. Alert of index case and cluster outbreak report

On 11 June 2021, an alert was generated by the China Information System for Disease Control and Prevention, regarding a laboratory-confirmed PTB student (the index case, case 1). Case investigation identified she was a 16-year-old student in class 16, grade 11. She first developed cough and expectoration in January 2021. After one week of ineffective anti-inflammatory treatment, which coincided with her final examinations, she deferred further medical care. Her symptoms persisted into the following semester and progressed to haemoptysis, prompting a hospital visit on 7 June 2021. Chest computed tomography (CT) revealed bilateral pulmonary nodules and patchy infiltrates suggestive of tuberculosis, and the diagnosis of PTB was confirmed by sputum smear microscopy on 11 June 2021.

**Figure 1.**
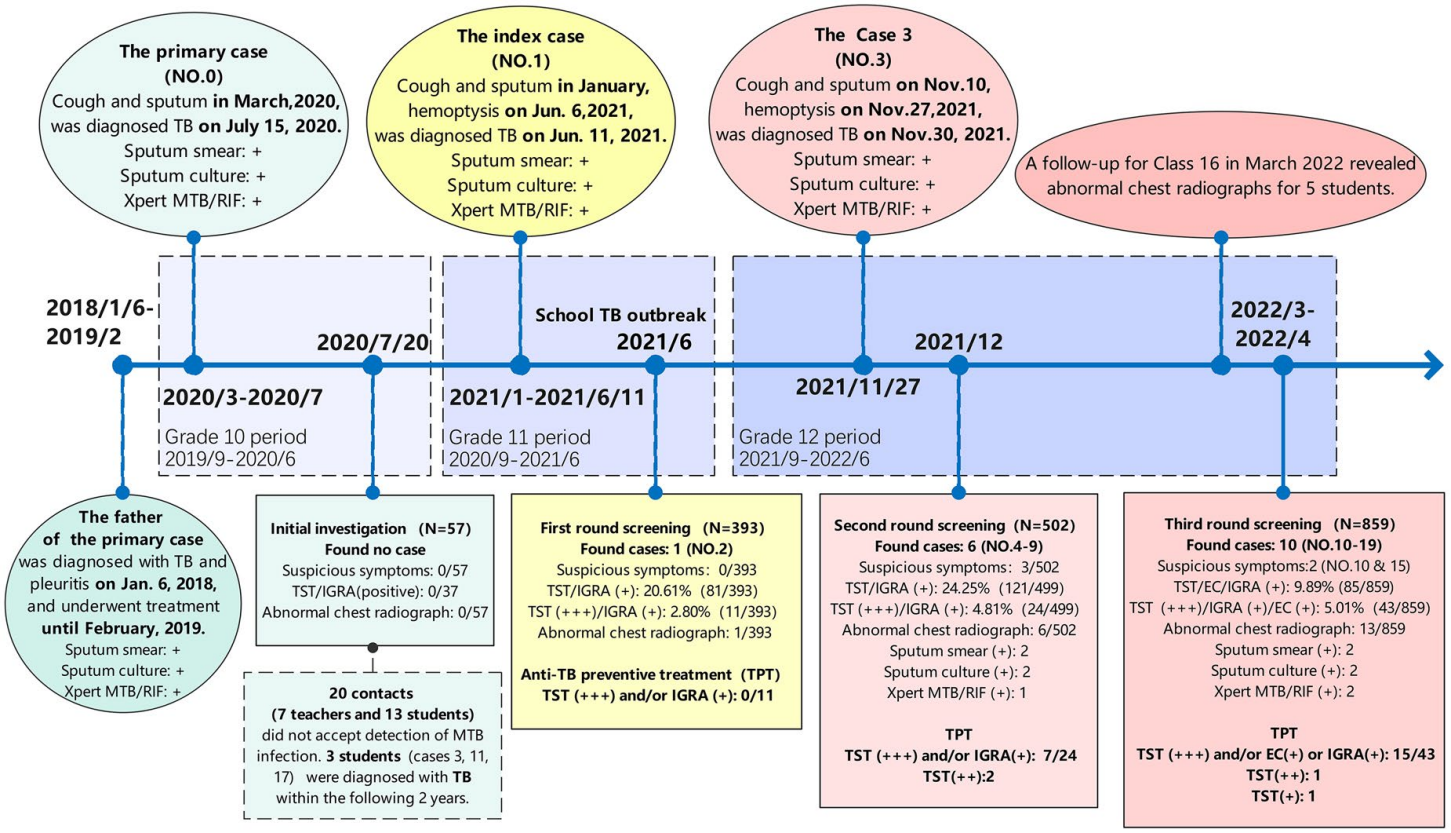
Timeline of the tuberculosis epidemic at a boarding senior high school in Eastern China, 2020–2022.

In response, the local county Center for Disease Control and Prevention (CDC) subsequently initiated the close contact screening on 14 June 2021 (Figure 1). Close contacts were defined as individuals sharing prolonged, confined-space exposure to the active PTB case, specifically staff and students from the same classroom and dormitory [11], which included 40 students and the head teacher from class 16. All screened individuals underwent concurrent symptom assessment, MTB infection testing, and chest radiography. MTB infection testing primarily employed the TST using purified protein derivative (PPD) with IGRA serving as an alternative for those contraindicated or who refused skin tests. Individuals presenting TB-suggestive symptoms, positive infection test results or abnormal chest imaging underwent pathogen testing via sputum analysed by Xpert MTB/RIF, culture and drug susceptibility testing [6]. The identification of case 2 (clinically PTB), the classmate and roommate of the index case, prompted a cluster outbreak report and expanded screening for general contacts defined as those sharing the same teaching or dormitory floor with the index case, comprising 368 students and 25 staff. No additional active TB cases were identified through this expanded screening effort. All 11 individuals eligible for tuberculosis preventive therapy (TPT) – based on a strongly positive PPD or positive IGRA result after ruling out active TB – declined the treatment. Consequently, they were scheduled for chest imaging follow-up at 3, 6 and 12 months post-initial screening, with referrals initiated for any abnormalities.

### 2.3. Subsequent cases and continuous screening

On 1 December 2021, a second contact screening was triggered by the reporting of case 3. He was a student from class 16 who needed to take several classes in class 1 every week and had tested TST general positive with no abnormalities on chest X-ray during the first-round screening in June. Upon recall, he developed a mild cough on around November 10. The symptoms progressed to haemoptysis by November 27, which prompted a hospital visit. A CT scan revealed nodular and patchy opacities in the left upper lobe and the right middle and lower lobes, with cavitation evident in the right lower lobe lesion. PTB with bronchial involvement was subsequently confirmed by a positive sputum smear on November 30.

The second-round screening initially targeted students and staff from class 16 and class 1. Following the detection of multiple suspected PTB cases by chest CT in class 16, the screening scope was expanded to include all individuals on the same floor. This round covered 448 students and 55 staff members and identified six new active PTB cases (cases 4–9). Of these, four were from class 16; one (case 6, class 13) had been a close contact of the index case in the preceding semester, and another (case 7, class 15) had been a close contact of case 3. In response to the escalating outbreak, the CDC extended TPT eligibility to individuals presenting a TST result of moderate positivity or greater without evidence of active TB disease (LTBI) [12]. Only eight students and one teacher accepted TPT. Those with a negative TST were required to undergo repeat testing after three months. Additionally, the students in class 16 were placed under enhanced health monitoring and scheduled for a follow-up chest X-ray after three months.

In March 2022, a follow-up for class 16 revealed five students with abnormal chest X-rays, and one additional student was diagnosed with PTB after presenting with cough and night sweats. In response, and capitalizing on the imminent National College Entrance Examination physicals, the local CDC conducted the third-round screening for the entire grade. To mitigate the potential impact of the TST boosting phenomenon on outbreak management, this round of screening primarily employed the TBST [10,13]. Only a few students on leave at home independently underwent TST at hospitals. Screening of 790 students and 67 teachers identified 10 active PTB cases (cases 10–19): eight in class 16, and one each in classes 13 and 15. Furthermore, 17 students received TPT; 13 of them were from class 16.

### 2.4. Integration of epidemiological and molecular evidence

**Figure 2.**
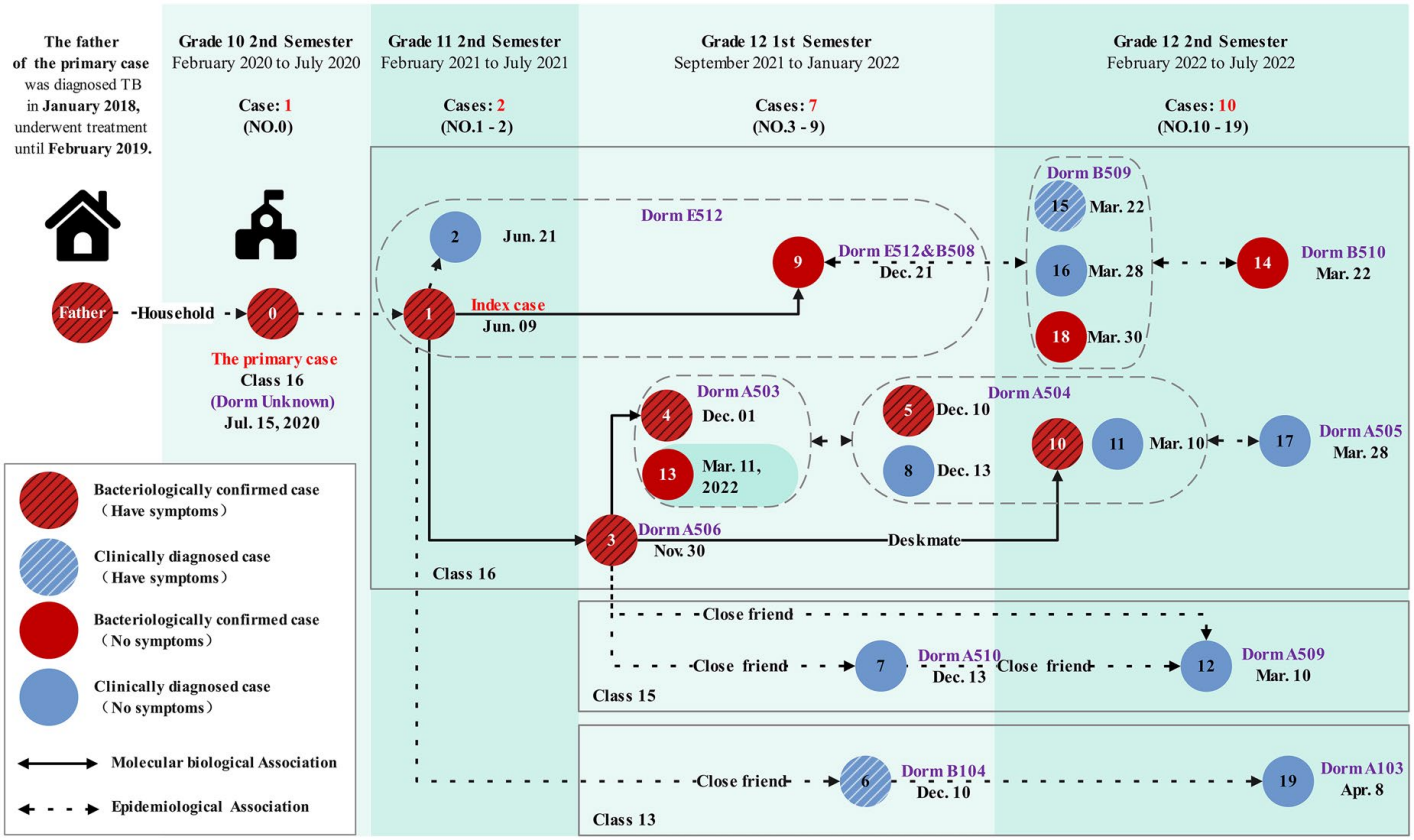
Epidemiologic and molecular biology network: links among cases in classrooms, dormitories and households.

Following comprehensive review and expert panel consultation, a total of 20 PTB cases were diagnosed among boarding students within the same grade, including 10 laboratory-confirmed and 10 clinical cases, eight of whom were symptomatic. The cases were distributed across classes 13, 15 and 16, involving boys’ dormitories A103, A503, A504, A505, A509 and A510, as well as girls’ dormitories B104, B508, B509, B510 and E512 (Figure 2).

Epidemiological investigation revealed no history of TB among family members or friends associated with the index case. All classmates having close contact with her developed disease only after her diagnosis, except for case 0, a previously reported PTB male student in class 16 in 2020. He had attended classes for over three months while symptomatic (cough and expectoration) and was diagnosed on 15 July 2020, after presenting with fever. Close contact tracing initiated at the time by the county CDC identified no secondary cases. However, the investigation encountered significant non-cooperation, as 20 close contacts (seven teachers and 13 students) refused TB infection testing (Figure 1). Notably, investigation revealed household exposure for case 0: his father had been diagnosed with PTB and tuberculous pleurisy in 2018 after a chronic cough lasting over a year. Further information indicated that case 10 had shared a desk with case 3. Key transmission pathways likely facilitating cross-class spread involved close contact between case 6 and case 1 combined with frequent exposure linking cases 7 and 12 to case 3, propagating infection from class 16 to classes 13 and 15.

Single nucleotide polymorphism (SNP) analysis was employed for mycobacterial genotyping and characterization of outbreak-associated MTB strains. Five strains of MTB were successfully isolated during this outbreak, including strains from the index case (case 1) and four classmates from class 16 in the following two semesters (cases 3, 4, 9 and 10). A maximum-likelihood phylogenetic tree was constructed using RAxML software [14] with *Mycobacterium canettii* (CIPT 140060008) as the root, and visualized with the ggtree package in R (Figure S1). Phylogenetic reconstruction revealed that all outbreak strains belonged to Lineage 2 (Beijing genotype). Pairwise genomic distances were compared; SNP analysis showed 0–12 SNP differences among the outbreak strains, confirming high genetic homology and supporting a clear transmission chain within class 16 in three semesters [15,16].

### 2.5. Detection of LTBI and active TB cases among contacts

By April 2022, three rounds of contact screening had been completed for the index case and subsequently identified cases. Attack rates and infection rates were calculated for categorical variables. Group differences were assessed using the chi-square test, with statistical significance defined as *p* < 0.05. The screenings identified 114 LTBI (12.94%) and 18 active TB cases (2.04%) (Table 1). The difference in the rate of LTBI between students and teachers was statistically significant (*χ*^2^ = 16.493, *p* < 0.001), whereas no significant difference was observed in the TB attack rate. Additionally, no significant difference was found in infection rates or case detection rates between male and female students. Furthermore, the infection rate among students in the index case class (class 16, 46.34%) was significantly higher than that in other case classes (classes 13 and 15 combined, 19.05%) and in classes with no reported cases (8.27%) (*χ*^2^ = 61.169, *p* < 0.001).

**Table 1.** Analysis on detection of LTBI and active TB cases among contacts (*N* = 881).

Group	*N*	LTBI				TB			
		*n*	Rate (%)	*χ* ^2^	*p* Value	*n*	Rate (%)	*χ* ^2^	*p* Value
Occupation									
Students	811	94	11.59	16.493	<0.001	18	2.34	0.671	0.413
Staff	70	20	28.57			0	0.00		
Total	881	114	12.94	–	–	18	2.04	–	–
Gender of students									
Male	392	51	13.01	1.492	0.222	10	2.55	0.436	0.509
Female	419	43	10.26			8	1.91		
Class									
16	41	19	46.34	61.169	<0.001	14	34.15	208.889	<0.001
13 and 15	105	20	19.05			4	3.81		
Other	665	55	8.27			0	0.00		

A review of tuberculosis infection testing revealed that although over half of cases showed positive results at diagnosis, 66.16% (12/19) had tested negative during prior screenings. It provided an update on the progression of the infections in these cases (Table 2).

**Table 2.** MTB infection status for cases in each round of screening (*N* = 19).

No.	Class	Initial investigation			First round			Second round			Third round			Laboratory examination results	Strain typing
		Method	Result	Mark	Method	Result	Mark	Method	Result	Mark	Method	Result	Mark		
1	16	TST	–		Untested	None	☑							SS(+), SC(+), S-Xpert(+)	Lineage 2
2	16	TST	–		TST	++	☑							SS(−), SC(−), S-Xpert(−)	
3	16	Refused	None		TST	+		TST	+	☑				SS(+), SC(+), S-Xpert(+)	Lineage 2
4	16	TST	–		TST	–		TST	–	☑				SS(−), SC(+), S-Xpert(−)	Lineage 2
5	16	TST	–		TST	+		TST	+	☑				SS(−), SC(−), S-Xpert(+)	
6	13	Excluded	None		TST	++		TST	+++	☑				SS(−), SC(−), S-Xpert(−)	
7	15	TST	–		TST	+		TST	–	☑				SS(−), SC(−), S-Xpert(−)	
8	16	TST	–		TST	+		TST	++	☑				SS(−), SC(−), S-Xpert(−)	
9	16	TST	–		TST	–		TST	++	☑				SS(−), SC(+), BAL-Xpert(+)	Lineage 2
10	16	TST	–		TST	–		TST	+		TST	+	☑	SS(−), SC(+), S-Xpert(+)	Lineage 2
11	16	Refused	None		TST	–		TST	–		TST	–	☑	SS(−), SC(−), S-Xpert(−)	
12	15	Excluded	None		TST	–		TST	–		TST	–	☑	SS(−), SC(−), S-Xpert(−)	
13	16	TST	–		TST	–		TST	–		TST	–	☑	SS(−), SC(+), S-Xpert(−)	
14	16	TST	–		TST	–		TST	–		TST	+++	☑	SS(+), SC(+), S-Xpert(+)	
15	16	TST	–		TST	–		TST	–		IGRA	+	☑	SS(−), SC(−), BAL-Xpert(−)	
16	16	TST	–		TST	–		TST	–		C–TST	+	☑	SS(−), SC(−), S-Xpert(−)	
17	16	Refused	None		TST	–		TST	–		C–TST	+	☑	SS(−), SC(−), S-Xpert(−)	
18	16	TST	–		TST	–		TST	–		IGRA	+	☑	SS(−), SC(−), S-Xpert(+)	
19	13	Excluded	None		TST	–		TST	–		TST	++	☑	SS(−), SC(−), S-Xpert(−)	

TST: tuberculin skin test; C-TST: creation tuberculin skin test; IGRA: interferon gamma release assay; (−) negative; (+) positive; (++) moderately positive; (+++) strongly positive; ☑ mark: the case was diagnosed in this screening round; SS: sputum smear; SC: sputum culture; S/BAL-Xpert: sputum/bronchoalveolar lavage fluid-Xpert MTB/RIF.

### 2.6. Follow-up monitoring outcomes

All 19 diagnosed cases, excluding case 0 who completed treatment pre-outbreak without returning to school, underwent medical withdrawal for tuberculosis treatment. Standard regimens followed the 2HRZE/4HRE protocol (Table S1). Among 26 individuals who received TPT, the 3HR regimen was prescribed, achieving a 78.95% (15/19) TPT coverage in class 16. Community healthcare workers and school clinic physicians jointly supervised medication adherence and monitored adverse reactions for both treatment and TPT groups. All participants completed their respective therapeutic courses. Following the third screening round, a one-year follow-up of the affected student population detected no new cases. To date, no additional TB cases have been reported through the ongoing follow-up of all household contacts of the confirmed patients.

## 3. Discussion

This incident exemplifies a characteristic multi-semester PTB cluster outbreak in a boarding high school in eastern China, speculating a community-origin transmission. Epidemiological investigation indicated that case 0, with a history of household exposure, was the most probable source of infection. SNP typing further confirmed transmission links among cases reported across multiple semesters within the school. As the outbreak developed, the screening scope progressively expanded from close contacts of infected individuals to all students and teachers in the affected grade. Intensive surveillance of class 16 facilitated the timely detection of multiple cases, and broadening preventive treatment played a key role in containing the spread. Throughout the response, long-term follow-up of both school and household contacts was maintained, ensuring effective outbreak control without community spillover. Additionally, longitudinal follow-up spanning nearly three years monitored LTBI progression and close contacts during their high school period, providing evidence on the development of active TB.

This outbreak demonstrated a community-to-school transmission pattern, evidenced by epidemiological links connecting case 0 to his father and subsequently to case 1. Household contact substantially elevates tuberculosis risk among adolescents in high-burden regions [17], where peak TB progression occurs within two years post-exposure [18]. Current Chinese community-based TB protocols limit follow-up monitoring to one year exclusively for contacts of laboratory-confirmed cases, failing to protect high-risk student populations. We recommend extending surveillance duration beyond two years while expanding monitoring to include contacts of clinically diagnosed cases, thereby enhancing early detection and mitigating cluster outbreak risks. Routine tuberculosis screening during enrolment demonstrates proven efficacy in preventing school-based cluster outbreaks [19,20]. Had such screening been implemented, high-risk individuals with TB exposure histories, such as case 0 who was a household contact of an active TB patient, would have been identified and prioritized for intervention, thereby potentially averting this outbreak.

SNP analysis demonstrated high genetic relatedness between the case 1 strain and strains from subsequent cases (cases 3, 4, 9 and 10), confirming sustained transmission within the school. Class 16 exhibited significantly elevated LTBI and TB case detection rates compared to other classes. This disparity likely resulted from prolonged exposure and pronounced respiratory symptoms in earlier cases including case 0 and case 1, underscoring that delayed case detection and isolation constituted the root cause of this epidemic. These findings align with established risk factors including inadequate TB control implementation, poor ventilation, prolonged exposure and insufficient TB prevention knowledge [4]. Daily health monitoring systems proved deficient. After diagnosis of case 0, twenty close contacts underwent chest imaging but refused MTB infection testing, creating potential transmission reservoirs. Dormitory clustering affected over half of cases, consistent with boarding school outbreak patterns [21], where symptomatic individuals in multi-case dormitories likely drove early transmission. Teachers showed higher LTBI rates than students, consistent with age-related progression patterns in China [22]. The lifetime risk of progression from LTBI to active TB is estimated at 5–10% among individuals without intervention, with incidence rates peaking within the first five years [23,24]. Current *the Guidelines* restrict TPT to individuals in school with strongly positive TST, positive IGRA or positive TBST [10,13]. Some scholars recommend expanding TPT to moderately TST-positive individuals during cluster outbreaks involving three or more cases or elevated regional LTBI rates, while also affirming that universal TPT remains warranted for all dormitory residents regardless of test results when housing symptomatic or laboratory-confirmed TB cases [25,26]. During this outbreak, 26 individuals completed the 3HR preventive regimen. Twenty-four of them exhibited strongly positive TST, positive IGRA or positive TBST. None experienced significant adverse drug reactions or developed active TB during follow-up. These outcomes underscore the necessity of educational interventions to enhance TPT acceptance and compliance for effective epidemic containment.

TB progression from initial infection to clinical onset generally unfolds over an extended timeframe. During the subclinical phase, although patients display minimal or no overt symptoms, MTB maintains metabolic activity within the host and may remain transmissible [27,28]. The diagnosis for active PTB relies on three kinds of laboratory evidence: microscopic detection of acid-fast bacilli, bacteriological culture and molecular biology methods (e.g. gene Xpert MTB/RIF) [29]. Notably, MTB sputum culture retains its status as the diagnostic gold standard and is critical for determining drug susceptibility profiles [30]. However, conventional diagnostic approaches frequently fail to detect MTB in suspected cases with scanty sputum or negative smears, particularly among children [31]. During this outbreak, sputum microscopy, culture and Xpert MTB/RIF testing failed to identify MTB in half of cases. Furthermore, several cases (e.g. case 6 and case 15) served as key transmission vectors despite their negative microbiological results, highlighting an urgent imperative to develop enhanced molecular diagnostics with superior sensitivity for timely confirmation of active tuberculosis. Wu et al. reported an 80% laboratory-confirmed case rate using bronchoscopically collected bronchoalveolar lavage (BAL) samples for Xpert MTB/RIF testing during a school TB outbreak [15], illustrating that BAL-based Xpert MTB/RIF detection enhances laboratory sensitivity and aids TB outbreak investigations. Additionally, although most cases demonstrated MTB infection at diagnosis, 66.16% of all individuals had previously tested negative during screening. This discrepancy may stem from the lack of a current gold standard for MTB infection detection, coupled with variability in diagnostic methods and host immune heterogeneity. Critically, these findings emphasize the necessity of ongoing monitoring for contacts with chronic exposure to laboratory-confirmed cases, even when initial MTB tests are negative, to enable timely preventive therapy and health monitoring following LTBI diagnosis.

Compared with previous epidemic investigations and responses [8], our approach to outbreak reporting and management was fundamentally similar, initiating close contact screening immediately upon reporting of the index case, and carrying out epidemic reporting and overall management based on updates of newly reported cases. However, in contrast, this outbreak spanned a longer duration, essentially covering the three-year high school period. In terms of the application of molecular epidemiological analysis, our bacterial strain samples were derived from cases reported across multiple semesters within the same class, representing longitudinal follow-up data. This contrasts with the cross-sectional findings reported by Yu et al. [7] and Wu et al. [15], and constitutes a distinctive feature of this study.

This study has several limitations. First, missing strain isolates restricted transmission pathway validation: case 0 and his father had completed treatment before outbreak investigation, precluding strain acquisition from these individuals and thereby preventing confirmation of community-to-school transmission via strain sequencing. Similarly, molecular bioinformatics analysis could not be performed for cases in classes 13 and 15 due to unavailable strains. Second, due to limitations in the survey data, we were unable to conduct a more in-depth analysis of infection risk. Specifically, the lack of quantitative data such as odds ratios for infection rates associated with different dormitory distances or levels of contact. Third, case reports spanning multiple semesters and frequent seating adjustments in class 16 hindered collection of original classroom seating arrangements, limiting inferences about intra-class transmission way. Finally, retrospective epidemiological investigations may be subject to recall bias.

In conclusion, this study concludes with an analysis of a multi-semester cluster PTB outbreak in a Chinese boarding school involving 20 cases and 114 LTBI. Key contributing factors included inadequate community-based contact tracing, insufficient TB screening for close contacts of the primary case, and a 6-month delay in diagnosing the index case. Molecular epidemiological associations among early cases across three semesters were confirmed through SNP typing. These findings underscore the critical importance of early detection, enhanced contact tracing and timely interventions in preventing and controlling TB outbreaks in school settings. Therefore, we recommend that in future outbreak responses, bronchoscopic examination be performed as early as possible for suspected cases, with molecular testing and culture of BAL conducted to facilitate rapid case identification. No new cases occurred during the three-year follow-up, though this may be coincidental. More outbreak reports with longer-term data are needed to better inform policy adjustments.

## Supplementary Material

supplementary figure and table.docx

IANN- open science form.docx

## Data Availability

The data supporting this study were obtained through outbreak investigation and laboratory analysis. Whole-genome sequencing data of the strains have been deposited in the National Microbiology Data Center under accession number NMDC10019837; however, certain restrictions apply. Part of the data was used under license for this study and is not publicly available. Data are available from the corresponding author upon reasonable request.

## Open scholarship

This article has earned the Center for Open Science badges for Open Data. The materials are openly accessible at https://nmdc.cn/resource/genomics/project/detail/NMDC10019837.
