# Supplementary material for: Responding to a protracted tuberculosis outbreak: lessons from multiple rounds of investigation in a Chinese boarding school
Source: Ann Med. 2026 Mar 2;58(1):2635885. doi: 10.1080/07853890.2026.2635885 (PMC12958379; doi:10.1080/07853890.2026.2635885)
Supplement: supplementary figure and table.docx [file IANN_A_2635885_SM5963.docx]

**Table S1 The demographic and baseline characteristics of the cases with PTB involved. (N=20)**

| NO. | Gender | Age | Class | Dormitory | Symptoms | Time of symptom onset | Detection of MTB  infection | Chest imaging  examination | Date of report | Laboratory examination results | Date of diagnosis | Diagnosis | Treatment |
| --- | --- | --- | --- | --- | --- | --- | --- | --- | --- | --- | --- | --- | --- |
| 0 | Male | 16 | 16 | unknown | Cough, sputum, fever | 2020/03 | None | CT abnormal | 2020/06/19 | SS(+), SC(+),  S-Xpert(+) | 2020/07/15 | Confirmed PTB | 2HRZE/4HR |
| 1 | Female | 17 | 16 | E512 | Cough,sputum, hemoptysis | 2021/01 | None | CT abnormal | 2021/06/07 | SS(+), SC(+),  S-Xpert(+) | 2021/06/11 | Confirmed PTB | 2HRZE/4HR |
| 2 | Female | 17 | 16 | E512 | No | × | TST(++) | CT abnormal | 2021/06/21 | SS(-), SC(-),  S-Xpert(-) | 2021/06/22 | Clinically PTB | 2HRZE/4HR |
| 3 | Male | 17 | 16 | A506 | Cough, sputum, hemoptysis | 2021/11/27 | TST(+) | CT abnormal | 2021/11/30 | SS(+), SC(+),  S-Xpert(+) | 2021/12/01 | Confirmed PTB,  Bronchial TB | 2HRZE/10HRE |
| 4 | Male | 18 | 16 | A503 | Cough | 2021/11/20 | TST(-) | CT abnormal | 2021/12/01 | SS(-), SC(+),  S-Xpert(-) | 2021/12/27 | Confirmed PTB | 2HRZE/4HRE |
| 5 | Male | 17 | 16 | A504 | Cough,  chest pain | 2021/11/15 | TST(+) | CT abnormal | 2021/12/10 | SS(-), SC(-),  S-Xpert(+) | 2021/12/10 | Confirmed PTB | 2HRZE/4HR |
| 6 | Female | 17 | 13 | B104 | Cough | 2021/11/20 | TST(+++) | CT abnormal | 2021/12/10 | SS(-), SC(-),  S-Xpert(-) | 2021/12/13 | Clinically PTB | 2HRZE/4HR |
| 7 | Male | 17 | 15 | A510 | No | × | TST(+) | CT abnormal | 2021/12/13 | SS(-), SC(-),  S-Xpert(-) | 2021/12/14 | Clinically PTB | 2HRZE/4HRE |
| 8 | Male | 18 | 16 | A504 | No | × | TST(++) | CT abnormal | 2021/12/13 | SS(-), SC(-),  S-Xpert(-) | 2021/12/15 | Clinically PTB | 2HRZE/4HRE |
| 9 | Female | 16 | 16 | E512&B508 | No | × | TST(++) | CT abnormal | 2021/12/21 | SS(-), SC(+),  BAL-Xpert(+) | 2021/12/22 | Confirmed PTB | 2HRZE/4HR |
| 10 | Male | 18 | 16 | A504 | Cough,  night sweats | 2022/03/06 | TST(+) | X-rays abnormal | 2022/03/10 | SS(-), SC(+),  S-Xpert(+) | 2022/03/10 | Confirmed PTB | 2HRZE/4HRE |
| 11 | Male | 17 | 16 | A504 | No | × | TST(-) | CT abnormal | 2022/03/10 | SS(-), SC(-),  S-Xpert(-) | 2022/03/10 | Clinically PTB | 2HRZE/4HRE |
| 12 | Male | 17 | 15 | A509 | No | × | TST(-) | CT abnormal | 2022/03/10 | SS(-), SC(-),  S-Xpert(-) | 2022/03/10 | Clinically PTB | 2HRZE/4HRE |
| 13 | Male | 17 | 16 | A503 | No | × | TST(-) | X-rays abnormal | 2022/03/11 | SS(-), SC(+),  S-Xpert(-) | 2022/03/11 | Confirmed PTB | 2HRZE/4HRE |
| 14 | Female | 18 | 16 | B510 | No | × | TST(+++) | X-rays abnormal | 2022/03/22 | SS(+), SC(+),  S-Xpert(+) | 2022/03/22 | Confirmed PTB | 2HRZE/4HRE |
| 15 | Female | 17 | 16 | B509 | Cough | 2022/03/10 | IGRA(+) | CT abnormal | 2022/03/22 | SS(-), SC(-),  BAL-Xpert(-) | 2022/03/24 | Clinically PTB | 2HRZE/4HRE |
| 16 | Female | 17 | 16 | B509 | No | × | C-TST(+) | CT abnormal | 2022/03/28 | SS(-), SC(-),  S-Xpert(-) | 2022/03/28 | Clinically PTB | 2HRZE/4HRE |
| 17 | Male | 18 | 16 | A505 | No | × | C-TST(+) | CT abnormal | 2022/03/28 | SS(-), SC(-),  S-Xpert(-) | 2022/03/28 | Clinically PTB | 2HRZE/4HRE |
| 18 | Female | 17 | 16 | B509 | No | × | IGRA(+) | CT abnormal | 2022/03/30 | SS(-), SC(-),  S-Xpert(+) | 2022/03/31 | Confirmed PTB | 2HRZE/4HRE |
| 19 | Male | 18 | 13 | A103 | No | × | TST(++) | CT abnormal | 2022/04/08 | SS(-), SC(-),  S-Xpert(-) | 2022/04/26 | Clinically PTB | 2HRZE/4HRE |

TST: tuberculin skin test (tuberculin purified protein derivative); C-TST: creation tuberculin skin test (*Mycobacterium tuberculosis* recombinant fusion protein ESAT6-CFP10); IGRA: Interferon gamma release assay; (-): negative; (+) positive; (++): moderately positive; (+++): strongly positive; CT: computed tomography; SS: Sputum smear; SC: Sputum culture; S/BAL-Xpert: Sputum/Bronchoalveolar lavage fluid-gene Xpert *Mycobacterium tuberculosis*/rifampin; H: isoniazid; R: rifampicin; Z: pyrazinamide; E: ethambutol.


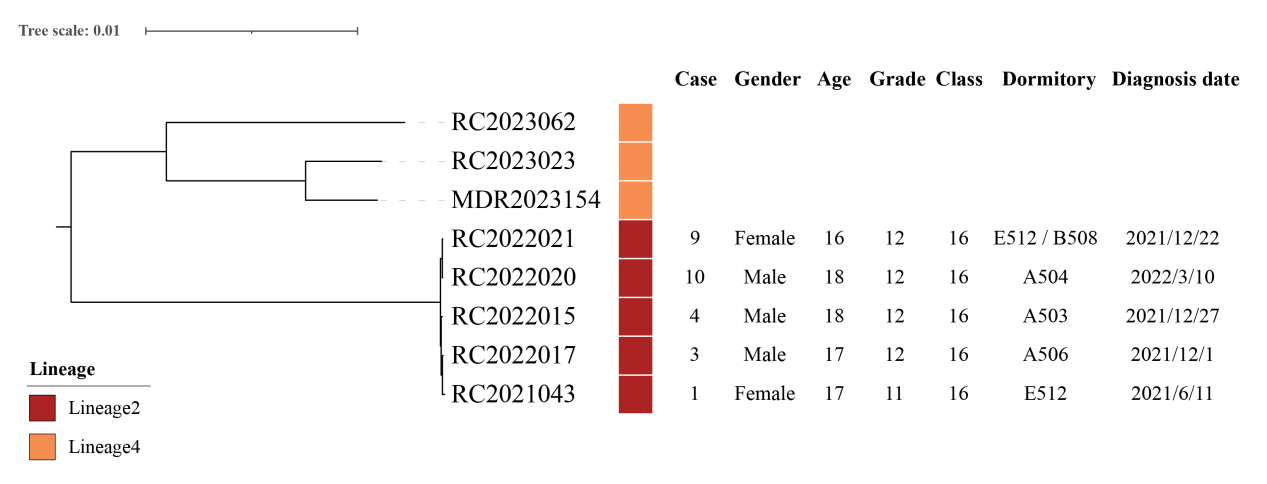


**Figure S1 Phylogenetic tree of Mycobacterium tuberculosis strains isolated from cases during this outbreak.**

Single Nucleotide Polymorphism (SNP) analysis was employed for mycobacterial genotyping and characterization of outbreak-associated MTB strains. Five strains of MTB were successfully isolated during this outbreak, including strains from the index case (Cases 1) and four classmates from Class 16 in the following two semesters (Cases 3, 4, 9, and 10). A maximum-likelihood phylogenetic tree was constructed using RAxML software with Mycobacterium canettii (CIPT 140060008) as the root, and visualized with the ggtree package in R (Figure S1).
